# Healthcare professionals’ knowledge of organic foods and their health impact: a survey-based analysis

**DOI:** 10.1016/j.clinsp.2025.100720

**Published:** 2025-08-05

**Authors:** Angela Cristine Bersch-Ferreira, Stela Verzinhasse Peres, Patrick Araujo Terezan, Gisele Medeiros Bastos, Rozana Mesquita Ciconelli, Fúlvio Alexandre Scorza

**Affiliations:** a Research Department, BP - A Beneficência Portuguesa de São Paulo, São Paulo, SP, Brazil; b Research and Education Department, Health Technology Assessment Center (NATS BP), BP - A Beneficência Portuguesa de São Paulo, São Paulo, SP, Brazil; c Department of Neurology and Neurosurgery, Universidade Federal de São Paulo, São Paulo, SP, Brazil

**Keywords:** Organic foods, Healthcare professionals, Synthetic fertilizers, Chemical pesticides

## Abstract

**Background:**

Organic foods are often considered healthier and more sustainable, yet healthcare professionals’ understanding and perceptions remain unclear. This study aimed to evaluate their knowledge and perceptions related to organic foods and associated barriers.

**Methods:**

A cross-sectional survey was conducted among employees at a large hospital in São Paulo, Brazil, between June and July 2024. Using a non-probabilistic convenience sample, an online questionnaire collected sociodemographic data, lifestyle characteristics, and information on participants’ knowledge and perception of organic foods. Descriptive statistics, Chi-Square tests, and logistic regression were applied to identify factors associated with the lack of knowledge.

**Results:**

A total of 199 questionnaires were completed. Nearly half of the respondents (49.2 %) were not fully aware of what defines organic food. Women represented 80.2 % of the sample. Smoking was significantly associated with a lack of knowledge regarding organic foods. Approximately 30 % were uncertain about cardiovascular risks related to non-organic foods, and cost was identified as a major barrier to consumption. Although most recognized environmental benefits and a potential role in reducing global warming, understanding how organic consumption intersects with human health has remained limited. Adjusted analyses revealed that higher education (without postgraduate training), smoking, and comorbidities were associated with knowledge gaps.

**Conclusion:**

These findings highlight substantial knowledge gaps among healthcare professionals regarding organic foods.

## Highlights

• Used a hospital-based survey to assess healthcare professionals' knowledge.
• Many lacked a clear definition of organic foods and their health benefits.
• High cost and uncertainty emerged as common consumption barriers.
• Enhanced education could boost evidence-based dietary guidance.

## Introduction

Organic foods are produced under strict certification standards that prohibit synthetic fertilizers, chemical pesticides, and Genetically Modified Organisms (GMOs), emphasizing sustainability through scientifically guided practices.^1^ These requirements ensure soil and water conservation, biodiversity preservation, and reduced pollution. Official seals confirm that the entire production chain ‒ from cultivation to processing ‒ meets these criteria. As a result, organic foods are not only free of chemical residues but also reflect a commitment to animal welfare, social equity, and natural resource conservation, setting them apart from conventional alternatives. This lack of clarity about what constitutes organic food can lead to a misunderstanding of the true benefits of its consumption, which extend far beyond simply avoiding pesticide exposure.

It is evident that conventional agricultural practices rely heavily on chemical fertilizers and pesticides, which are widely recognized for their harmful effects on both human health and the environment. The widespread use of pesticides, particularly in countries like Brazil, has raised significant concerns due to their association with acute and chronic health issues, including dermatological, respiratory, cardiovascular, and neuropsychiatric effects.^2^, ^3^, ^4^, ^5^, ^6^, ^7^ As such, many experts advocate for viewing the use of pesticides in Brazil as a public health emergency, given the broad population exposure, from workers in pesticide production and agricultural fields to consumers of contaminated food.^8^ As public awareness about these issues grows, consumers are increasingly seeking food products that not only offer high nutritional value and sensory appeal but also provide additional health benefits.^9^, ^10^, ^11^

Besides being free of pesticides, organic foods are also environmentally safe, as they involve fewer chemical pesticides and avoid harmful methods like irradiation, industrial solvents, or synthetic additives in their production processes.^10^ This perception aligns with broader consumer trends favoring products that are viewed as natural and environmentally friendly. Additionally, organic farming emphasizes ecological sustainability and animal welfare, which further distinguishes it from conventional farming methods.^12^

Given the potential health and environmental benefits of organic foods, it is critical to assess the public’s understanding of these products, particularly among healthcare professionals, who play a key role in educating the population about healthy consumption habits. Understanding the level of knowledge and motivation among healthcare providers regarding organic food and pesticide risks is essential for developing effective public health interventions. This study seeks to assess healthcare professionals' knowledge of organic foods, identify key barriers to their consumption, and explore how sociodemographic and lifestyle factors may influence knowledge levels.

## Materials and methods

This is a cross-sectional survey, employing a non-probabilistic convenience sample. This study was reported in accordance with the STROBE Statement. The protocol was approved by the Research Ethics Committee of Hospital Beneficência Portuguesa de São Paulo (CAAE: 78363324.6.0000.5483). The survey was conducted among employees from a hospital in São Paulo, Brazil, with dissemination via Workplace and email between June and July 2024. An email was sent to all employees across various departments of the hospital and medical specialties, including administrative staff, consultants, and outsourced professionals

The study was approved by the Local Research Ethics Committee and conducted in accordance with national and international resolutions on good clinical research practices. All participants provided electronic consent to participate in the study. Upon accessing the survey link, participants were presented with an invitation to participate and the Informed Consent Form (ICF). A contact phone number and email address of the research team were provided to address any questions before deciding to participate. Participants also received a copy of the ICF via email. Data were collected only after the participant's consent.

To be considered an eligible response, the questionnaire had to be completed by an employee (regardless of whether they worked directly with patients or in administrative roles). Participation in the survey was voluntary, and no questions were mandatory. Participants had the option to withdraw from the survey at any point and could pause and return to the questionnaire later within the same session.

The data collection system used for the survey was REDCap, a secure web-based platform with various functionalities, including participant registration, data cleaning, and exporting data for statistical analysis. To ensure confidentiality, the survey was completed anonymously through the electronic platform (REDCap), without collecting any personal identifiers such as names, emails, or registration numbers. No login or institutional credentials were required to access the survey, and participants could complete it freely without their responses being linked to their identity. All data were stored on a secure server with restricted access, and analyses were conducted using only de-identified, aggregated data. The survey consisted of 26 questions divided into two sections. The first section focused on characterizing the population, collecting sociodemographic information such as age, gender, ethnicity, city, state, education level, and income, as well as lifestyle factors, including physical activity, smoking habits, alcohol consumption, and comorbidities. The authors defined comorbidities as the self-report of at least one chronic health condition (hypertension, diabetes, hyperlipidemia, psychiatric disorder, or neurological disorder).

The second section was specifically designed to assess participants' knowledge of the benefits of organic food, their consumption habits, and their access to organic foods. Completing the survey was estimated to take approximately 10 minutes.

The electronic survey forms were accessed via a link generated by the REDCap system. Missing data were not imputed; individuals with missing values were excluded from the analysis related to the specific variable with missing data. Since this study involved professionals from the institution, the authors prioritized ensuring that participants did not feel obligated to respond or uncomfortable when answering. Therefore, the authors opted for anonymous responses. As a result, it was not possible to track whether an individual submitted the questionnaire more than once, and no mechanism was in place to identify unique visitors. Data entry was subject to various checks, including open fields, plausible value ranges, valid and invalid inputs, as well as logical checks. Participants were notified of any issues during data entry. Descriptive analysis was conducted using absolute and relative frequencies, measures of central tendency, and dispersion. To assess the association between categorical variables and the outcome, knowledge about organic foods, the Chi-Square test or Fisher’s Exact test was applied when an expected cell count was less than or equal to five.

Univariate binary logistic regression analysis and unconditional multiple binary logistic regression analysis were performed to obtain Odds Ratios (aOR) with 95 % Confidence Intervals (95 % CIs). Covariates with significant p-values (< 0.050) and those with p-values < 0.200 were tested in the multiple regression model. A manual selection technique was used, considering variables in order from the lowest to the highest p-value. Confounding and interaction factors were assessed during the modeling process. The final model was built based on the following criteria: 1) No change in ORs greater than 10 %; 2) Improved accuracy as reflected by the 95 % CI; 3) Total degrees of freedom allowed for each outcome variable; and 4) Quality of the final model, assessed using the Hosmer-Lemeshow test. Multiple Correspondence Analysis (MCA) was used to analyze and identify patterns of association between categorical variables. The technique applied was factor analysis, aimed at identifying the main axes (dimensions) of variation. Data analysis was performed using RStudio, version 4.1.2.

## Results

### Demographic characteristics

A total of 199 questionnaires were completed. Among the respondents, 62 (49.2 %) reported not knowing what an organic food is. Most were women (80.2 %), with a mean age of 37.8 years (SD = 9.0), a median of 37 years, and an age range of 19 to 63. The sample included both administrative and assistance staff, reflecting the hospital’s diverse range of professional roles. Table 1 compares the characteristics of participants who were knowledgeable versus those who were unaware of what constitutes an organic food. Although the majority held administrative positions (69.4 %), there were no statistically significant differences in knowledge levels between administrative and assistance professionals (Table 1). Smoking was significantly associated with a lack of knowledge about organic foods. Although participants lacking this knowledge had higher frequencies of hypertension, hypercholesterolemia, and psychological disorders, these differences were not statistically significant.

**Table 1:** Number and percentage of participants by demographic, lifestyle characteristics, and comorbidities.

Variables	Category	Total		What is an organic food?				p^a^
				Unaware		Know		
		n	%	n	%	n	%	
Age range (median)	< 37	92	47.7	62	49.2	30	44.8	0.557
	≥ 37	101	52.3	64	50.8	37	55.2	
Sex	Female	158	80.2	103	79.8	55	80.9	0.862
	Male	39	19.8	26	20.2	13	19.1	
What job do you do at BP?	Administrative	136	69.4	86	67.7	50	72.5	0.491
	Assistance	60	30.6	41	32.3	19	27.5	
Education	≤ High school	25	12.8	16	12.6	9	13.0	0.089
	Higher education	138	70.4	95	74.8	43	62.3	
	Postgraduate	33	16.8	16	12.6	17	24.6	
Have a partner	No	94	47.5	64	49.6	30	43.5	0.410
	Yes	104	52.5	65	50.4	39	56.5	
People/household	1 and 2	82	41.2	53	41.1	29	41.4	0.931
	2 to 3	88	44.2	58	45.0	30	42.9	
	4 or more	29	14.6	18	14.0	11	15.7	
What is the monthly income range of the family (R$)?	Up to 3000	15	7.5	12	9.3	3	4.3	0.440
	3001‒10,000	105	52.8	67	51.9	38	54.3	
	> 10,000	79	39.7	50	38.8	29	41.4	
Smoker	No	175	88.4	109	84.5	66	95.7	***0.020***
	Yes	23	11.6	20	15.5	3	4.3	
Acoholic	No	147	73.9	97	75.2	50	71.4	0.564
	Yes	52	26.1	32	24.8	20	28.6	
Nutritional status	Eutrophic	77	39.3	46	36.5	31	44.3	0.392
	Overweight	79	40.3	51	40.5	28	40.0	
	Obese	40	20.4	29	23.0	11	15.7	
Do you have any comorbidities?	No	162	81.4	101	78.3	61	87.1	0.126
	Yes	37	18.6	28	21.7	9	12.9	
Diabetes (any type)	No	188	97.9	120	97.6	68	98.6	1.000
	Yes	4	2.1	3	2.4	1	1.4	
Hypertension	No	180	91.4	113	89.0	67	95.7	0.107
	Yes	17	8.6	14	11.0	3	4.3	
Hyperlipidemia	No	178	93.2	113	91.9	65	95.6	0.386^b^
	Yes	13	6.8	10	8.1	3	4.4	
Psychiatric disorder	No	181	94.8	115	93.5	66	97.1	0.499^b^
	Yes	10	5.2	8	6.5	2	2.9	
Neurological disorder	No	189	99.5	121	99.2	68	100.0	1.000^b^
	Yes	1	0.5	1	0.8	0	0.0	

a Chi-Square.

b Fisher’s Exact test.

### Knowledge of organic foods

Participants were also asked about the health and environmental aspects related to organic food consumption (Table 2). Approximately 30 % were unable to determine whether consuming non-organic foods could lead to cardiovascular harm. Regarding health benefits, 74.2 % were aware of them, although 12.6 % were uncertain. In terms of environmental impact, 15.2 % expressed uncertainty, yet nearly 80 % considered that consuming organic foods could mitigate negative effects on global warming.

**Table 2:** Number and percentage of participants by perceived harms and benefits of organic foods.

Variables	Category	Total		What is an organic food?				p^a^
				Unaware		Know		
		n	%	n	%	n	%	
Do you believe that pesticides and/or chemical fertilizers harm health?	No	4	2.0	2	1.6	2	2.9	0.077^b^
	Yes	188	94.5	120	93.0	68	97.1	
	Unable to answer	7	3.5	7	5.4	0	0.0	
Cancer	No	4	2.0	2	1.6	2	2.9	0.214^b^
	Yes	178	89.4	113	87.6	65	92.9	
	Unable to answer	17	8.5	14	10.9	3	4.3	
Cardiovascular disease	No	20	10.1	9	7.0	11	15.7	0.138
	Yes	118	59.3	78	60.5	40	57.1	
	Unable to answer	61	30.7	42	32.6	19	27.1	
Skin diseases	No	10	5.0	6	4.7	4	5.7	0.891
	Yes	148	74.4	97	75.2	51	72.9	
	Unable to answer	41	20.6	26	20.2	15	21.4	
Eye problems	No	26	13.1	14	10.9	12	17.1	0.347
	Yes	87	43.7	60	46.5	27	38.6	
	Unable to answer	86	43.2	55	42.6	31	44.3	
Problem with the nervous system/brain (neurological disease)	No	12	6.0	8	6.2	4	5.7	0.935
	Yes	136	68.3	89	69.0	47	67.1	
	Unable to answer	51	25.6	32	24.8	19	27.1	
Respiratory disease	No	17	8.5	14	10.9	3	4.3	0.163
	Yes	128	64.3	84	65.1	44	62.9	
	Unable to answer	54	27.1	31	24.0	23	32.9	
Kidney diseases	No	12	6.0	8	6.2	4	5.7	0.929^b^
	Yes	148	74.4	97	75.2	51	72.9	
	Unable to answer	39	19.6	24	18.6	15	21.4	
Gastric problems	No	5	2.5	4	3.1	1	1.4	0.697^b^
	Yes	164	82.4	107	82.9	57	81.4	
	Unable to answer	30	15.1	18	14.0	12	17.1	
Problems during pregnancy (Fetus)	No	14	7.0	11	8.5	3	4.3	0.561^b^
	Yes	138	69.3	87	67.4	51	72.9	
	Unable to answer	47	23.6	31	24.0	16	22.9	
Are you aware of the health benefits of organic foods for human health?	No	26	13.1	17	13.3	9	12.9	0.995
	Yes	147	74.2	95	74.2	52	74.3	
	Unable to answer	25	12.6	16	12.5	9	12.9	
Prevents several diseases	No	15	10.3	13	14.0	2	3.8	0.146^b^
	Yes	117	80.7	71	76.3	46	88.5	
	Unable to answer	13	9.0	9	9.7	4	7.7	
Ensures the health and safety of the family	No	6	4.2	5	5.4	1	2.0	0.691^b^
	Yes	129	89.6	82	88.2	47	92.2	
	Unable to answer	9	6.3	6	6.5	3	5.9	
Provides more nutrients	No	12	8.3	8	8.6	4	7.7	0.894^b^
	Yes	119	82.1	75	80.6	44	84.6	
	Unable to answer	14	9.7	10	10.8	4	7.7	
Contains elements, such as pesticides. that confer harm	No	3	2.1	2	2.2	1	1.9	1.000^b^
	Yes	131	90.3	84	90.3	47	90.4	
	Unable to answer	11	7.6	7	7.5	4	7.7	
Are you aware of the environmental benefits of organic foods?	No	32	16.2	18	14.1	14	20.0	0.233
	Yes	136	68.7	87	68.0	49	70.0	
	Unable to answer	30	15.2	23	18.0	7	10.0	
Reduction of water pollution	No	1	0.7	0	0.0	1	2.0	0.133^b^
	Yes	132	97.1	86	98.9	46	93.9	
	Unable to answer	3	2.2	1	1.1	2	4.1	
Conservation of biodiversity and environment	No	1	0.7	1	1.2	0	0.0	1.000^b^
	Yes	131	97.0	83	96.5	48	98.0	
	Unable to answer	3	2.2	2	2.3	1	2.0	
Lower impact on global warming	No	8	5.9	6	6.9	2	4.1	0.554^b^
	Yes	108	79.4	70	80.5	38	77.6	
	Unable to answer	20	14.7	11	12.6	9	18.4	
Protection of animal health	No	2	1.5	1	1.1	1	2.0	0.785^b^
	Yes	132	97.1	84	96.6	48	98.0	
	Unable to answer	2	1.5	2	2.3	0	0.0	
Encourages family farming and small producers	No	3	2.2	2	2.3	1	2.0	0.868^b^
	Yes	125	92.6	80	93.0	45	91.8	
	Unable to answer	7	5.2	4	4.7	3	6.1	

a Chi-square.

b Fisher’s Exact test.

Multiple logistic regression analysis identified education level, smoking, and comorbidities as independent factors associated with a lack of knowledge about organic foods (Table 3). Adjusted for age, individuals with higher (but not postgraduate) education were more likely to lack knowledge compared to those with postgraduate degrees (aOR = 2.66; 95 % CI 1.19–5.95). Smokers had approximately four times the odds of lacking knowledge (aOR = 4.42; 95 % CI 1.16–15.47), while participants with comorbidities had about twice the odds (aOR = 2.54; 95 % CI 1.02–6.33).

**Table 3:** Factors associated with the lack of awareness about organic foods.

Model^a^	aOR	95 % CI		p
		Lower	Upper	
**Education**				
Postgraduate	Reference			
≤ High school	1.35	0.44	4.18	0.597
Higher education	2.66	1.19	5.95	*0.017*
**Smoker**	4.24	1.16	15.47	*0.029*
**Do you have any comorbidities?**	2.54	1.02	6.33	*0.046*

a Quality of model according to Hosmer-Lemeshow test = 0.896; adjusted model by age.

To provide further insight into the factors associated with knowledge about organic foods, the authors conducted two sets of univariate binary logistic regression analyses. Supplementary Table 1 reports the crude odds ratios for sociodemographic and lifestyle variables, including education level, smoking status, and presence of comorbidities. Supplementary Table 2 explores the associations between participants’ perceptions of health and environmental risks related to non-organic foods and their knowledge levels. These supplementary analyses support and expand upon the main findings, reinforcing the observed trends in knowledge gaps among healthcare professionals.

Multiple Correspondence Analysis (MCA) revealed that the first two dimensions accounted for less than 50 % of the total variance when considering all eligible variables among participants familiar and unfamiliar with organic food concepts. The results (Supplementary Fig. 1a–c) indicate that although the most relevant dimensions ‒ Dimension 1 (12.77 %) and Dimension 2 (8.53 %) ‒ were the most significant, together they explained only 21.3 % of the total variance. Dimensions 1 through 5 accounted for 43.06 % of the variance, representing less than half of the overall information content. Notably, 14 dimensions were required to reach a cumulative variance of 84.8 %, suggesting a highly complex and multifactorial data structure.

Key findings include the low explanatory power of individual dimensions, with none accounting for more than 12.8 % of the variance, indicating a poorly structured dataset characterized by multiple independent categories and weak inter-category associations. Furthermore, the high dimensionality, with 21 dimensions required to account for 100 % of the variance, suggests that the variance was broadly distributed and implies the presence of predominantly non-linear relationships among variables.

### Barriers to consumption

**Fig. 1:**
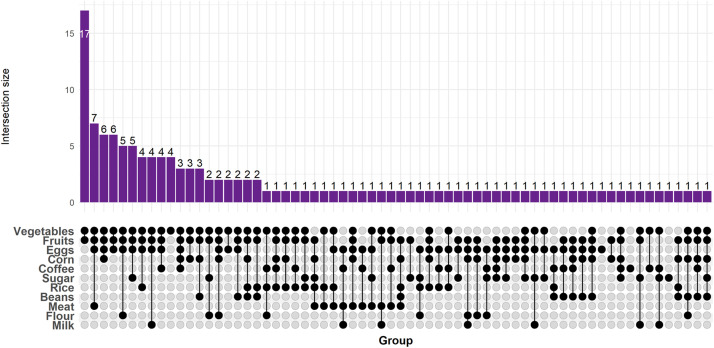
Distribution of organic food consumption.

Among participants who consumed organic foods (*n* = 132), fruits and vegetables were the most frequently mentioned items, followed by eggs and meats (Fig. 1).

When asked about their consumption habits, 33.7 % stated they did not consume organic foods, while 23.6 % did so at least once a week. Of those who purchased organic foods, 62.3 % obtained them from street markets or specialty stores. High cost was identified as the main barrier to consumption (27.6 %) (Table 4).

**Table 4:** Number and percentage of participants according to reasons for not consuming organic foods.

Variables	Category	Total		What is an organic food?				p^a^
				Unaware		Know		
		n	%	n	%	n	%	
Do you consume organic foods?	No	67	33.7	43	33.3	24	34.3	0.383
	A few times a month	85	42.7	59	45.7	26	37.1	
	At least once a week	47	23.6	27	20.9	20	28.6	
Where are organic foods purchased?^c^	Street markets or directly from the producer	49	37.7	33	38.4	16	36.4	0.823
	Markets/ Specialty stores	81	62.3	53	61.6	28	63.6	
I believe/understand the benefits	Yes	190	95.5	123	95.3	67	95.7	1.000^b^
	No	9	4.5	6	4.7	3	4.3	
The cost is very high	No	144	72.4	93	72.1	51	72.9	0.908
	Yes	55	27.6	36	27.9	19	27.1	
I do not find the product easily	No	172	86.4	112	86.8	60	85.7	0.828
	Yes	27	13.6	17	13.2	10	14.3	
What sources of information do you use to learn about organic foods?	Friends/ Relatives	27	13.6	16	12.4	11	15.7	0.909
	Internet/ Social networks	102	51.3	66	51.2	36	51.4	
	Newspaper/ TV/ Magazines	45	22.6	30	23.3	15	21.4	
	Others	25	12.6	17	13.2	8	11.4	
Where are organic foods purchased?	Street markets or directly from the producer	49	37.7	33	38.4	16	36.4	0.823
	Markets/ Specialty stores	81	62.3	53	61.6	28	63.6	

a Chi-Square.

b Fisher’s Exact test.

c Valid values for participants who reported consuming organic foods.

## Discussion

The present study aimed to examine the level of knowledge regarding the concept of organic foods among healthcare professionals in a large hospital in São Paulo, Brazil. Nearly half of the participants (49.2 %) were not fully aware of what constitutes an organic food. Given that these individuals operate in a healthcare environment, where the relationship between diet, health, and disease prevention should be more evident, this finding highlights a critical need for more effective dissemination of information on organic foods. The association between smoking and a lack of knowledge suggests that certain lifestyle factors may reflect or influence overall health awareness among these professionals.

Organic foods are not merely pesticide-free; they adhere to strict certification standards that ensure sustainable soil and water management, prohibit Genetically Modified Organisms (GMOs), and uphold animal welfare.^13^ Nonetheless, the present results point to a knowledge gap extending beyond simple recognition of the “organic” label. For instance, approximately 30 % of participants were uncertain whether non-organic foods could pose cardiovascular risks. This uncertainty echoes the complexity noted in a systematic review,^14^ which found insufficient evidence to draw definitive conclusions on the health benefits of organic diets. Although about 80 % acknowledged the role of organic consumption in mitigating global warming, understanding how ecological factors intersect with human health remains an area in need of reinforcement. Enhanced educational efforts could bridge these gaps, empowering healthcare professionals to act as informed agents of change, disseminating evidence-based information on the health benefits of organic foods and the importance of sustainable practices.

In terms of nutrition and disease prevention, the perceived advantages of organic foods often include their higher concentrations of certain beneficial compounds such as antioxidants, particularly polyphenols,^15^ and increased levels of omega-3 fatty acids in organic dairy products.^16^, ^17^, ^18^ Nevertheless, concerns linger about the long-term implications of pesticide residues found in conventional foods. While consumers generally recognize the environmental and health advantages of organic foods,^19^^,^^20^ research has shown that this awareness does not necessarily translate into informed decision-making. The present findings suggest that even among healthcare professionals, more robust and targeted educational initiatives are needed to clarify the complex relationships between environmental sustainability, human health, and organic food production.

This notion is further supported by studies employing frameworks like the Theory of Planned Behavior, which emphasize the importance of knowledge in shaping attitudes and intentions.^21^^,^^22^ As these studies highlight, greater knowledge of organic foods correlates with more positive intentions and behaviors. Improving educational strategies ‒ through formal training, readily accessible resources, or public health campaigns ‒ could enable professionals to more effectively communicate both the health-related and ecological implications of organic food consumption, thus enhancing their role as advocates for sustainable dietary choices.

The literature also indicates that factors such as high prices, limited availability, and irregular supply restrict organic food consumption.^19^ In the present study, participants similarly identified cost as an obstacle and demonstrated limited understanding of what organic foods entail. While previous research has noted minimal behavioral differences between men and women, the focus lies in the broader need to expand education among health professionals of all genders. By enhancing awareness of both the health and environmental benefits of organic foods, these professionals can be better equipped to guide others toward healthier, more sustainable dietary choices.

A plausible explanation for inconsistent organic food consumption among healthcare professionals may lie in the interplay of limited knowledge and the higher costs associated with organic foods. As indicated by the present findings and supported by other studies, uncertainty about the nature and health implications of organic foods can deter more informed choices.^1^^,^^2^ The perception of high prices ‒ often tied to production, distribution, and certification challenges ‒ has long been noted as a barrier to increasing consumption.^3^ In settings where individuals are uncertain about the tangible benefits, paying more for organic foods may seem unjustified, perpetuating underconsumption rooted in both economic and informational constraints.

Despite these barriers, the global organic market is experiencing substantial growth, with approximately 80 billion euros (92 billion USD) spent annually on organic foods, and a 14.7 % increase in organic farmland worldwide.^23^^,^^24^ This trend is mirrored in Brazil, where both the number of certified organic producers and the amount of organic farmland have expanded in recent years.^25^ As consumers become increasingly aware of health and environmental benefits, the flourishing organic sector presents opportunities to foster more cost-effective strategies and enhance access. By clarifying the value of organic foods and promoting supportive policies, healthcare professionals can contribute to integrating these products into regular diets, ultimately benefiting both public health and the environment.

Importantly, the present findings have practical implications for both institutional practices and public health policies. As healthcare professionals are key players in promoting healthy eating habits, the observed knowledge gaps support the implementation of educational strategies within hospitals, such as awareness campaigns and continuous training. Beyond the hospital setting, these results may guide broader public health initiatives, informing policies that combine access to organic foods with professional education and helping shape more sustainable and equitable food systems.

One strength of this study is the inclusion of a diverse group of healthcare professionals, providing a valuable perspective on how individuals with varying roles and backgrounds perceive and understand organic foods. The use of a validated Electronic Data Collection Platform (REDCap) and the anonymous nature of the responses likely reduced social desirability bias. Conducting the research within a hospital setting, inherently focused on health, adds practical context to these findings. However, certain limitations must be considered. The non-probabilistic convenience sample, the absence of a formal sample size calculation, and the fact that the study was conducted in a single hospital may limit the generalizability of the findings. Although participants came from different departments and job functions, the results may not reflect the knowledge and perceptions of healthcare professionals in other institutions or regions of Brazil. Future multicenter studies with larger and more representative samples are recommended to confirm these findings and provide broader insights. Additionally, the inability to track unique participants raises the possibility of duplicate responses, and the cross-sectional design precludes causal inferences. Moreover, self-reported data are subject to recall bias or inaccuracies in self-assessment. There is also a possibility of social desirability bias, as participants, being healthcare professionals, might overreport their knowledge or positive attitudes toward organic foods. Additionally, since participation was voluntary, there may be a self-selection bias favoring individuals who already have some interest in the topic. These factors must be considered when interpreting the results, as they may influence the accuracy and representativeness of the reported knowledge and perceptions.

The present findings are consistent with other studies conducted among healthcare professionals and consumers. For example, a multinational study identified that nutritional knowledge among healthcare workers in several European countries was often insufficient, highlighting the need for improved professional training and continuing education.^26^ Similarly, another study emphasized that while organic foods are generally perceived as healthier by the public, understanding of their actual health effects remains limited, suggesting a gap between perception and scientific evidence.^27^ These findings reinforce the need for targeted educational initiatives focused on both nutritional and environmental aspects of food systems.

In addition, a recent article provided a comprehensive review of the nutritional value and health impacts of organic foods, noting potential benefits such as reduced exposure to chemical residues and improved antioxidant profiles, although evidence remains inconclusive in some areas.^28^ These findings support the relevance of improving awareness and scientific understanding among healthcare professionals, as observed in this study.

Furthermore, a recent study analyzed the profiles and perceptions of organic food producers and consumers, identifying that high cost and limited availability continue to be important barriers to consumption, even among those who recognize potential health benefits. These barriers were also cited by participants in the present study, reinforcing the need for intersectoral policies to improve access to organic foods in Brazil.^29^

Despite these constraints, these results underscore the need for educational interventions and more robust research designs to elucidate how healthcare professionals can best influence organic food consumption practices, ultimately guiding their patients and communities toward healthier, more sustainable dietary patterns.

## Conclusion

In this study, nearly half of the healthcare professionals surveyed lacked a clear understanding of what defines an organic food, revealing a critical knowledge gap in a group expected to promote healthy dietary habits. While most recognized potential benefits, uncertainties about health impacts and environmental implications persisted, and perceived barriers like cost and limited access further hindered confident recommendations to patients.

These findings underscore the importance of continuous professional education on food systems, sustainability, and nutrition. Without foundational knowledge, healthcare professionals are limited in their ability to guide the public toward healthier, more sustainable food choices. Public health policies should also support initiatives that clarify the definition and benefits of organic foods, ensuring professionals are equipped to act as effective advocates. Future studies with more robust designs and broader samples are essential to inform these strategies.

## Declaration of generative AI and AI-assisted technologies in the writing process

During the preparation of this work, the authors used ChatGPT to improve language. After using this tool, the authors reviewed and edited the content as needed and take full responsibility for the content of the publication.

## Funding

This research did not receive any specific grant from funding agencies in the public, commercial, or not-for-profit sectors.

## Declaration of competing interest

The authors declare no conflicts of interest.

## Appendix Supplementary materials

Image, application 1
