# Supplementary material for: Healthcare professionals’ knowledge of organic foods and their health impact: a survey-based analysis
Source: Clinics (Sao Paulo). 2025 Aug 5;80:100720. doi: 10.1016/j.clinsp.2025.100720 (PMC12375199; doi:10.1016/j.clinsp.2025.100720)
Supplement: Supplementary file 1 [file mmc1.docx]

**CLINICS-D-25-00175**

**Supplementary Materials**

Univariate binary logistic regression analysis (Supplementary Table 1 and 2) and unconditional multiple binary logistic regression analysis were performed to obtain Odds Ratios (aOR) with 95% Confidence Intervals (95% CIs). Covariates with significant p-values (< 0.050) and those with p-values <0.200 were tested in the multiple regression model. A manual selection technique was used, considering variables in order from the lowest to the highest p-value. Confounding and interaction factors were assessed during the modeling process. The final model was built based on the following criteria: 1) No change in ORs greater than 10%; 2) Improved accuracy as reflected by the 95% CI; 3) Total degrees of freedom allowed for each outcome variable; and 4) Quality of the final model, assessed using the Hosmer-Lemeshow test. Data analysis was performed using RStudio, version 4.1.2.

**Supplementary Table 1** Univariate binary logistic regression.

| **Variables and categories** | **OR** | **CI 95%** | | **p** |
| --- | --- | --- | --- | --- |
|  |  | **Lower** | **Upper** |  |
| Age (≥ 37/ < 37) | 0.84 | 0.46 | 1.52 | 0.558 |
| Sex (male/female) | 1.07 | 0.51 | 2.24 | 0.862 |
| What job do you do at BP? (Assistance/Administrative) | 1.25 | 0.66 | 2.39 | 0.491 |
| Education (Postgraduate) | Ref |  |  |  |
| ≤ High school | 1.89 | 0.65 | 5.48 | 0.242 |
| Higher education | 2.35 | 1.08 | 5.08 | 0.030 |
| Have a partner (Yes/No) | 0.78 | 0.43 | 1.41 | 0.411 |
| People/household (1 and 2) | Ref |  |  |  |
| 2 to 3 | 1.06 | 0.56 | 1.99 | 0.861 |
| 4 or more | 0.90 | 0.37 | 2.15 | 0.805 |
| What is the monthly income range of the family (R$)? (up to 3,000) | Ref |  |  |  |
| 3,001‒10,000 | 0.44 | 0.12 | 1.66 | 0.226 |
| > 10,000 | 0.43 | 0.11 | 1.65 | 0.220 |
| Smoker (Yes/No) | 4.04 | 1.15 | 14.11 | 0.029 |
| Alcoholic (Yes/No) | 0.82 | 0.43 | 1.59 | 0.564 |
| Nutritional status (eutrophic) | Ref |  |  |  |
| overweight | 1.23 | 0.64 | 2.35 | 0.535 |
| obese | 1.78 | 0.77 | 4.08 | 0.175 |
| Do you have any comorbidities? (Yes/No) | 1.88 | 0.83 | 4.25 | 0.130 |
| Diabetes (any type) ‒ (Yes/No) | 1.70 | 0.17 | 16.66 | 0.649 |
| Hypertension (Yes/No) | 2.77 | 0.77 | 9.98 | 0.120 |
| Hyperlipidemia (Yes/No) | 1.92 | 0.51 | 7.22 | 0.336 |
| Psychiatric disorder (Yes/No) | 2.30 | 0.47 | 11.13 | 0.302 |

**Supplementary Table 2** Univariate binary logistic regression.

| **Variables and categories** | **OR** | **95% CI** | | **p** |
| --- | --- | --- | --- | --- |
|  |  | **Lower** | **Upper** |  |
| Do you believe that pesticides and/or chemical fertilizers harm health? (No) | Ref |  |  |  |
| Yes | 1.76 | 0.24 | 12.81 | 0.574 |
| Unable to answer | xx | xx | xx |  |
| Cancer (Yes) | Ref |  |  |  |
| No | 0.58 | 0.08 | 4.18 | 0.585 |
| Unable to answer | 2.68 | 0.74 | 9.69 | 0.132 |
| Cardiovascular disease (Yes) | Ref |  |  |  |
| No | 0.42 | 0.16 | 1.10 | 0.076 |
| Unable to answer | 1.13 | 0.58 | 2.20 | 0.711 |
| Skin diseases (No) | Ref |  |  |  |
| Yes | 1.27 | 0.34 | 4.70 | 0.722 |
| Unable to answer | 1.16 | 0.28 | 4.76 | 0.841 |
| Eye problems (No) | Ref |  |  |  |
| Yes | 1.90 | 0.78 | 4.66 | 0.158 |
| Unable to answer | 1.52 | 0.63 | 3.70 | 0.355 |
| Problem with the nervous system/brain (neurological disease) | Ref |  |  |  |
| Yes | 0.95 | 0.27 | 3.31 | 0.932 |
| Unable to answer | 0.84 | 0.22 | 3.18 | 0.800 |
| Respiratory disease (Yes) | Ref |  |  |  |
| No | 2.44 | 0.67 | 8.96 | 0.178 |
| Unable to answer | 0.71 | 0.37 | 1.35 | 0.295 |
| Kidney diseases (No) | Ref |  |  |  |
| Yes | 0.95 | 0.27 | 3.31 | 0.937 |
| Unable to answer | 0.80 | 0.20 | 3.13 | 0.748 |
| Gastric problems (No) | Ref |  |  |  |
| Yes | 0.47 | 0.05 | 4.30 | 0.503 |
| Unable to answer | 0.38 | 0.04 | 3.78 | 0.405 |
| Problems during pregnancy (Fetus) ‒ (No) | Ref |  |  |  |
| Yes | 1.89 | 0.46 | 7.77 | 0.376 |
| Unable to answer | 0.88 | 0.44 | 1.76 | 0.720 |
| Are you aware of the health benefits of organic foods for human health? (Yes) | Ref |  |  |  |
| Yes | 0.97 | 0.40 | 2.32 | 0.940 |
| Unable to answer | 0.94 | 0.30 | 2.97 | 0.918 |
| Prevents several diseases (Yes) | Ref |  |  |  |
| No | 4.21 | 0.91 | 19.53 | 0.066 |
| Unable to answer | 1.46 | 0.42 | 5.01 | 0.550 |
| Ensures the health and safety of the family (No) | Ref |  |  |  |
| Yes | 0.35 | 0.04 | 3.08 | 0.343 |
| Unable to answer | 0.40 | 0.03 | 5.15 | 0.482 |
| Provides more nutrients (No) | Ref |  |  |  |
| Yes | 0.85 | 0.24 | 2.99 | 0.803 |
| Unable to answer | 1.25 | 0.24 | 6.63 | 0.793 |
| Are you aware of the environmental benefits of organic foods? (No) | Ref |  |  |  |
| Yes | 1.38 | 0.63 | 3.02 | 0.418 |
| Unable to answer | 2.56 | 0.85 | 7.65 | 0.094 |
| Lower impact on global warming (No) | Ref |  |  |  |
| Yes | 0.61 | 0.12 | 3.19 | 0.562 |
| Unable to answer | 0.41 | 0.07 | 2.53 | 0.335 |
| Protection of animal health (No) | Ref |  |  |  |
| Yes | 1.75 | 0.11 | 28.62 | 0.695 |
| Unable to answer | xx | xx | xx |  |
| Encourages family farming and small producers (No) | Ref |  |  |  |
| Yes | 0.89 | 0.08 | 10.08 | 0.924 |
| Unable to answer | 0.67 | 0.04 | 11.29 | 0.779 |

Note: xx, Categories for which the values were zero.

**Supplementary Figure 1 (A‒C) Multiple Correspondence Analysis.** Multiple Correspondence Analysis (MCA) was conducted to explore the relationships among categorical variables in relation to knowledge about organic foods. The first two dimensions explained 21.3% of the total variance (12.77% and 8.53%, respectively), and five dimensions together accounted for only 43.06% of the variance. A total of 14 dimensions were needed to explain 84.8% of the variance, indicating a complex, multifactorial structure with weak associations among categories and a high degree of data dispersion.
